# Green fluorescent protein-based lactate and pyruvate indicators suitable for biochemical assays and live cell imaging

**DOI:** 10.1038/s41598-020-76440-4

**Published:** 2020-11-11

**Authors:** Kazuki Harada, Takami Chihara, Yuki Hayasaka, Marie Mita, Mai Takizawa, Kentaro Ishida, Mary Arai, Saki Tsuno, Mitsuharu Matsumoto, Takeshi Ishihara, Hiroshi Ueda, Tetsuya Kitaguchi, Takashi Tsuboi

**Affiliations:** 1 Department of Life Sciences, Graduate School of Arts and Sciences, The University of Tokyo, 3-8-1 Komaba, Meguro, Tokyo 153-8902 Japan; 2 Department of Biological Sciences, Graduate School of Science, The University of Tokyo, 7-3-1 Hongo, Bunkyo, Tokyo 113-0033 Japan; 3 Myoridge Co. Ltd., 46-29 Yoshidashimoadachi-cho, Sakyo, Kyoto 606-8501 Japan; 4 Department of Biology, Faculty of Science, Kyushu University, 774 Motooka, Nishi, Fukuoka 819-0395 Japan; 5 Dairy Science and Technology Institute, Kyodo Milk Industry Co. Ltd., Hinode-machi, Nishitama-gun, Tokyo 190-0182 Japan; 6 Laboratory for Chemistry and Life Science, Institute of Innovative Research, Tokyo Institute of Technology, 4259 Nagatsuta-cho, Midori-ku, Yokohama, Kanagawa 226-8503 Japan

**Keywords:** Fluorescence imaging, Cellular imaging

## Abstract

Glycolysis is the metabolic pathway that converts glucose into pyruvate, whereas fermentation can then produce lactate from pyruvate. Here, we developed single fluorescent protein (FP)-based lactate and pyruvate indicators with low EC_50_ for trace detection of metabolic molecules and live cell imaging and named them “Green Lindoblum” and “Green Pegassos,” respectively. Green Lindoblum (EC_50_ of 30 µM for lactate) and Green Pegassos (EC_50_ of 70 µM for pyruvate) produced a 5.2- and 3.3-fold change in fluorescence intensity in response to lactate and pyruvate, respectively. Green Lindoblum measured lactate levels in mouse plasma, and Green Pegassos in combination with D-serine dehydratase successfully estimated D-serine levels released from mouse primary cultured neurons and astrocytes by measuring pyruvate level. Furthermore, live cell imaging analysis revealed their utility for dual-colour imaging, and the interplay between lactate, pyruvate, and Ca^2+^ in human induced pluripotent stem cell-derived cardiomyocytes. Therefore, Green Lindoblum and Green Pegassos will be useful tools that detect specific molecules in clinical use and monitor the interplay of metabolites and other related molecules in diverse cell types.

## Introduction

Nearly all eukaryotic organisms use glucose as an energy resource. Glucose is catabolised within cells by glycolysis and further utilised in the citric acid cycle and electron transport chain to produce ATP. Pyruvate is the end product of glycolysis and serves as an important intermediate metabolite for carbohydrate homeostasis. Under aerobic conditions, pyruvate is mainly utilised in the citric acid cycle for oxidative phosphorylation. In contrast, under anaerobic conditions, pyruvate is converted into lactate or ethanol by fermentation^1^. Ineffective glycolysis or lactate metabolism due to pyruvate dehydrogenase complex deficiency or lactate dehydrogenase subunit deficiency causes severe metabolic disorders. Thus, it is important to understand the mechanisms of glycolysis and lactate metabolism in live cells at a high spatial and temporal resolution. Förster resonance energy transfer (FRET)-type lactate (Laconic) and pyruvate (Pyronic) indicators have been used to observe the intracellular dynamics of lactate and pyruvate^2,3^ and are useful in monitoring the intracellular glycolytic dynamics. However, FRET-type indicators are difficult to apply for multi-colour imaging in single cells as the indicator must acquire the fluorescence at two different wavelengths to visualise one molecule. While recent studies have succeeded in developing multiplex FRET measurements^4,5^, it requires careful choice of fluorescent protein (FP) pairs and precise equipment to avoid spectral crosstalk. Single FP-based indicators can visualise the dynamics of target molecules with a single wavelength of fluorescence and are promising for observing other molecules or in different organelles.

We previously developed single FP-based red, green, and blue ATP indicators (MaLions) and green glucose indicator (Green Glifon) for imaging in combination with other probes^6,7^. Recently, a single FP-based pyruvate indicator (EC_50_ ~ 480 µM, dynamic range 2.5-fold) successfully monitored pyruvate dynamics both in cultured cells and ex vivo^8^. However, EC_50_ values may still be improved to detect subtle changes in pyruvate levels. For example, under various conditions including starvation, intracellular pyruvate level is lower than the detection range of previously developed indicators (Pyronic: EC_50_ 107 µM)^3^.

Here, we developed single green FP-based lactate and pyruvate indicators with lower EC_50_ values and named them as “Green Lindoblum” (**Green** **L**actate **ind**icat**o**r suita**b**le for f**lu**orescence i**m**aging) and “Green Pegassos” (**Green** **P**yruvat**e** sensin**g** **a** **s**ingle fluore**s**cent pr**o**tein-ba**s**ed probe), respectively. Green Lindoblum and Green Pegassos enabled biochemical measurement of lactate and D-serine, respectively, and clearly revealed intracellular lactate and pyruvate kinetics in living cells. We propose that these indicators will aid and contribute to multi-colour imaging of glycolytic dynamics.

## Results and discussion

### Development of single FP-based lactate and pyruvate indicators Green Lindoblum and Green Pegassos

**Figure 1:**
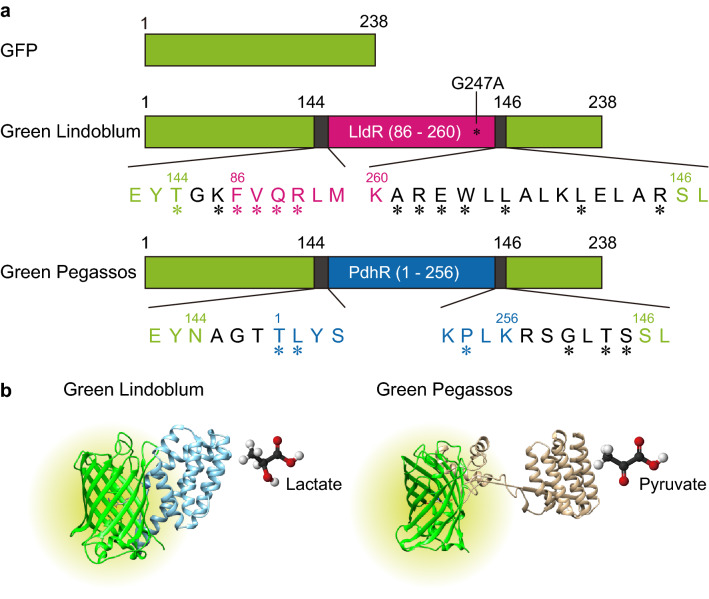
Schematic design of Green Lindoblum and Green Pegassos. (**a**) Diagrams for green fluorescent protein (GFP), Green Lindoblum, and Green Pegassos. Asterisks indicate mutations. (**b**) Schematic 3-D images of Green Lindoblum and Green Pegassos. Images were created using structural graphics for GFP (PDB_2Y0G) and *Escherichia coli* lactate dehydrogenase transcriptional regulator (LldR) or pyruvate dehydrogenase transcriptional regulator (PdhR). Three-dimensional models of LldR and PdhR were based on structural simulation by M4T Server 3.0 in Dr. András Fiser’s Laboratory (https://www.fiserlab.org/servers_table.htm)^26^.

We developed single FP-based indicators for lactate and pyruvate by inserting the *Escherichia coli* lactate binding domain of lactate dehydrogenase transcriptional regulator (LldR, WP_001297449.1, amino acids 86–260) or the DNA binding domain plus the pyruvate binding domain of pyruvate dehydrogenase transcriptional regulator (PdhR, NP_414655.1) into the vicinity of the chromophore of GFP. On the basis of our previous work^9^, we designed various prototype constructs with different lengths of linker peptides based on leucine zipper sequences and mutated amino acid residues between the ligand-binding domain and GFP. Following the screening, we obtained variants with the largest increase of fluorescence intensity in the presence of lactate and pyruvate (Fig. 1, Supplementary Fig. S1, S2, and S3).

### Spectral properties of Green Lindoblum and Green Pegassos, and measurement of estimation of lactate and D-serine levels

**Figure 2:**
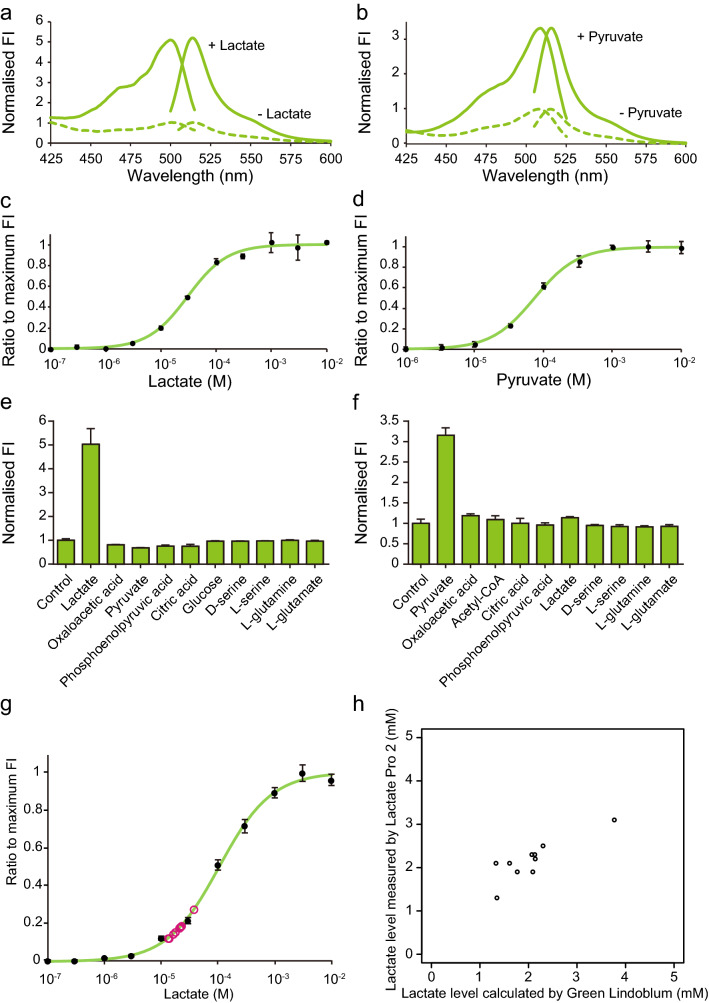
Spectral properties of Green Lindoblum and Green Pegassos. (**a**,**b**) Excitation and emission spectra of Green Lindoblum in the presence (solid line) and absence (dashed line) of 10 mM lactate (**a**), and Green Pegassos in the presence (solid line) and absence (dashed line) of 1 mM pyruvate (**b**). The fluorescence intensity (FI) was normalised to the peak in the absence of lactate or pyruvate. (**c**,**d**) Dose-response curve of Green Lindoblum and Green Pegassos. DR, dynamic range. (**e**,**f**) The specificity of Green Lindoblum and Green Pegassos to various glucose metabolism-related molecules (3 mM for Green Lindoblum and 700 µM for Green Pegassos). The peak FI for each metabolite was normalised to the peak FI in the distilled water (control). The data are shown as means ± standard deviation (n = 3). (**g**) Calibration of plasma lactate levels based on the dose-response curve of Green Lindoblum. The fluorescence intensity (FI) of Green Lindoblum with a 100-fold dilution of mouse plasma was plotted (magenta circles) to calculate the lactate level. The dose-response curve data are acquired in PBS aside from those in (**c**), and shown as means ± standard deviation (n = 3). (**h**) Correlation between the lactate levels calculated by Green Lindoblum and those measured by Lactate Pro 2. Pearson’s correlation coefficient, R = 0.8511, *P* = 0.00179.

We then investigated the spectral properties of Green Lindoblum and Green Pegassos using these recombinant proteins. Green Lindoblum had an excitation peak at 500 nm and an emission peak at 514 nm and increased 5.2-fold in fluorescence intensity with the addition of 10 mM lactate (Fig. 2a). Green Pegassos had an excitation peak at 504 nm and an emission peak at 516 nm and had a 3.3-fold increase in fluorescence intensity when 1 mM pyruvate was added (Fig. 2b). Based on dose-response curves and four-parameter logistic curve fitting, the half maximal effective concentration (EC_50_) of Green Lindoblum and Green Pegassos were 30 µM and 70 µM, respectively (Fig. 2c,d). The EC_50_ values of Green Lindoblum and Green Pegassos are lower than previously developed indicators^2,3,8^, and therefore, they would be appropriate to monitor changes in the minimal level of intracellular lactate and pyruvate. Hill coefficient for Green Lindoblum and Green Pegassos was 1.2 and 1.4, respectively, suggesting that these indicators are applicable for an appropriate range of lactate and pyruvate levels.

Green Lindoblum and Green Pegassos increased in FI with pH elevation both in the presence and absence of lactate or pyruvate (Supplementary Fig. S4). This generally occurs with single FP-based indicators and requires careful interpretation of the effect of pH changes when using these indicators for live cell imaging experiments. Furthermore, we investigated the ligand specificity of Green Lindoblum and Green Pegassos by adding various glucose metabolism-related molecules. Both indicators showed a minimal change of FI with other molecules (Fig. 2e,f), indicating Green Lindoblum and Green Pegassos were sufficiently specific to analyse the dynamics of metabolic molecules.

To take advantage of the low EC_50_ of Green Lindoblum and Green Pegassos, we then assessed them in further biochemical assays. First, we measured blood lactate levels in mice and compared the data with a commercial blood lactate test meter (Lactate Pro 2). We added 1/100-diluted mouse plasma samples to Green Lindoblum protein and calculated the lactate levels based on the dose-response curve (Table 1). These results correlated well to those from the Lactate Pro 2 test (R = 0.8511, *P* = 0.00179, Fig. 2g,h). Furthermore, we collected the samples from mice before and after lactate tolerance test^10^. Both Lactate Pro 2 and Green Lindoblum reported an increase of lactate levels 10 min after intraperitoneal injection of 1 g/kg lactate, showing that Green Lindoblum can distinguish changes in blood lactate levels (Supplementary Fig. S5).

**Table 1:** Measurement of plasma lactate using Green Lindoblum.

Sample No	Lactate level calculated by Green Lindoblum (mM)	Lactate level measured by Lactate Pro 2 (mM)
1	2.30	2.5
2	1.33	2.1
3	2.09	1.9
4	3.77	3.1
5	2.07	2.3
6	1.35	1.3
7	2.14	2.2
8	1.77	1.9
9	2.13	2.3
10	1.61	2.1

We next attempted to indirectly quantify other molecules related to lactate and pyruvate. D-serine acts as a co-agonist for N-methyl-D-aspartate glutamate receptors and is produced primarily in astrocytes and released as a gliotransmitter to modulate neurotransmission in the brain^11,12^. To quantify D-serine levels, previous studies used D-serine dehydratase (Dsd), which dehydrates D-serine to produce pyruvate and ammonia^13,14^, and also used lactate dehydrogenase to calculate the amount of degraded pyruvate from the absorbance of NADH. We attempted to directly measure the amount of pyruvate with Green Pegassos and, thereby, calculate D-serine levels. We mixed D-serine, Dsd, pyridoxal monophosphate, and Green Pegassos, and acquired a dose-response curve based on the FI of Green Pegassos (Supplementary Fig. S6). We measured D-serine levels in the supernatant of artificial cerebrospinal fluid (aCSF) from mouse primary cultured neurons and astrocytes after treatment in various conditions. D-serine could be detected from samples treated for 1 h with allyl isothiocyanate (AITC), which is known to induce D-serine release^15^, or serotonin, a major neurotransmitter (Table 2). However, it was difficult to detect D-serine from control samples incubated for 1 h; a further hour (2 h) of incubation allowed us to calculate D-serine levels (0.0512 mM, 0.0611 mM, 0.0596 mM). Thus, we propose that Green Pegassos is a useful tool for detecting not only their target molecules but also other related molecules in combination with enzymes, such as D-serine from various biological samples in vitro. However, given that we were unable to detect D-serine from control samples with short incubation, further improvement on sampling and measurement procedures will be necessary.

**Table 2:** Measurement of D-serine released from mouse primary cultured neurons and astrocytes using Green Pegassos.

Condition	Sample no	D-serine (mM)
Control (aCSF) 1 h	1	0.05
	2	ND
	3	ND
300 µM AITC 1 h	1	0.12
	2	0.07
	3	0.09
1 mM Serotonin 1 h	1	0.08
	2	0.09
	3	0.04

### Live cell imaging with Green Lindoblum and Green Pegassos

**Figure 3:**
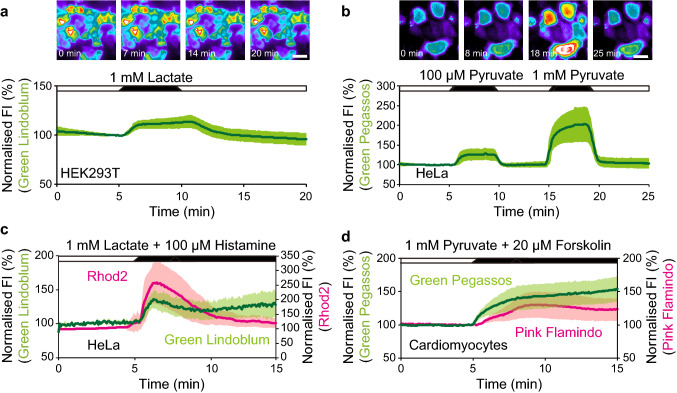
Live cell imaging using Green Lindoblum and Green Pegassos. (**a**,**b**) Top, sequential pseudo-colour images of HEK293T cells expressing Green Lindoblum (**a**) or HeLa cells expressing Green Pegassos (**b**). Scale bar represents 30 µm. Bottom, time courses of fluorescence intensity (FI) in the cells during the application of 1 mM lactate (**a**) or 100 µM and 1 mM pyruvate (**b**). (**c**) Time courses of FI of Green Lindoblum (green line) and Rhod2 (red line) in HeLa cells during the application of 1 mM lactate and 100 µM histamine. (**d**) Time courses of FI of Green Pegassos (green line) and Pink Flamindo (red line) in human iPS cell-derived cardiomyocytes during the application of 30 mM glucose. The data are shown as means ± standard deviation (n = 85 cells from five independent experiments (**a**), 28 (**b**), 35 (**c**) and 23 (**d**) cells from three independent experiments). The RB without stimuli was perfused as shown in white bars.

We then validated the utility of Green Lindoblum and Green Pegassos in living cells by applying lactate to Green Lindoblum-expressing HEK293T cells and pyruvate to Green Pegassos-expressing HeLa cells, respectively. Cells were imaged in modified Ringer’s buffer (RB), and the FI of both Green Lindoblum and Green Pegassos showed a reversible increase upon stimulation, despite the smaller dynamic ranges compared to in vitro experiments (Green Lindoblum: 114.1 ± 5.5% to 1 mM lactate, Green Pegassos: 129.0 ± 15.0% to 100 µM pyruvate and 204.9 ± 44.5% to 1 mM pyruvate, Fig. 3a, b, Supplementary Fig. S7a to e). When compared to previously developed FRET-based indicators Laconic and Pyronic, Green Lindoblum showed a larger response to 1 mM lactate than Laconic, and Green Pegassos showed a larger response to both 100 µM and 1 mM pyruvate than Pyronic (Laconic: 109.3 ± 4.2% to 1 mM lactate, Pyronic: 103.1 ± 1.5% to 100 µM pyruvate and 128.8 ± 4.8% to 1 mM pyruvate, Supplementary Fig. S7f to j). These results suggest that Green Lindoblum and Green Pegassos are applicable for observing real-time dynamics of intracellular lactate and pyruvate. Even though there are drawbacks for the use of these indicators due to pH sensitivity and limited quantification compared to FRET-based indicators, it is worthwhile to introduce Green Lindoblum and Green Pegassos for more experimental opportunities which otherwise could not be suited for FRET imaging.

We also monitored the response of plasma membrane-localised Green Lindoblum and Green Pegassos (Green Lindoblum-CAAX and Green Pegassos-CAAX, respectively, Supplementary Fig. S8a,b). Both Green Lindoblum-CAAX and Green Pegassos-CAAX showed an increase in the FI in response to lactate and pyruvate, respectively (Green Lindoblum-CAAX: 108.9 ± 5.9% to 1 mM lactate, Green Pegassos-CAAX: 144.1 ± 7.2% to 100 µM pyruvate and 180.4 ± 20.7% to 100 µM pyruvate, Supplementary Fig. S8c to i).

Next, we took advantage of single FP-based indicators and performed dual-colour imaging with Green Lindoblum or Green Pegassos, and the red Ca^2+^-sensitive dye, Rhod2. When HeLa cells expressing Green Lindoblum and loaded with Rhod2 were exposed to 1 mM lactate and 100 µM histamine, the FI of both Green Lindoblum and Rhod2 increased (Green Lindoblum: 144.5 ± 15.4%, Rhod2: 266.7 ± 72.3%, Fig. 3c, Supplementary Fig. S9a to c). Histamine activates histamine receptors, which are coupled to G_q_ proteins, thereby causing Ca^2+^ mobilisation from the endoplasmic reticulum^16^, which corresponds with our results. Furthermore, we expressed Green Lindoblum and the red cAMP indicator Pink Flamindo in cardiomyocytes derived from human induced pluripotent stem (iPS) cells. Application of 1 mM pyruvate and 20 µM forskolin (adenylate cyclase activator) induced an increase in the FI of both Green Pegassos and Pink Flamindo (Green Pegassos: 154.8 ± 19.0%, Pink Flamindo: 132.8 ± 20.1%, Fig. 3d, Supplementary Fig. S9d to f). Taken together, Green Lindoblum and Green Pegassos are applicable to dual-colour imaging in various cell types.

### Measurement of glycolytic dynamics in human iPS cell-derived cardiomyocytes

**Figure 4:**
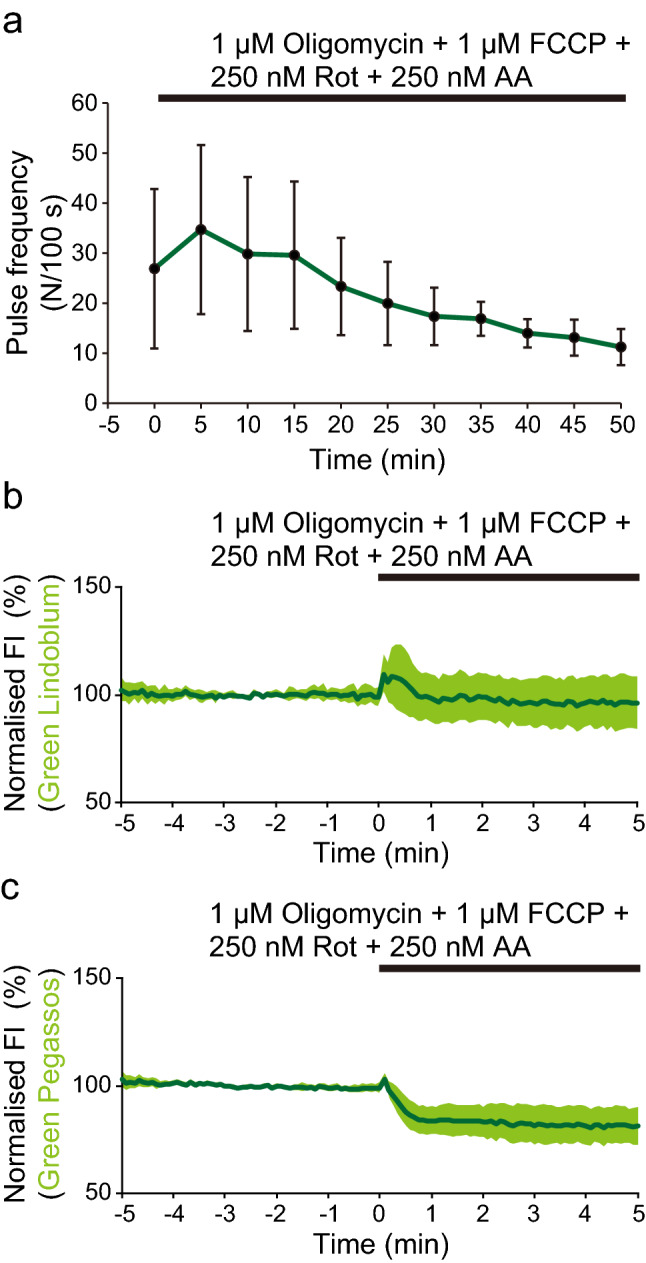
Imaging with human iPS cell-derived cardiomyocytes. (**a**) Pulse frequency following the combined application of 1 μM oligomycin, 1 μM FCCP, 250 nM Rot, and 250 nM AA (n = 17 cells from four independent experiments). (**b**,**c**) Time courses of fluorescence intensity (FI) of Green Lindoblum (**b**) and Green Pegassos (**c**) immediately after the combined application of inhibitors. The data are shown as means ± standard deviation (n = 13 (**b**) and 14 (**c**) cells from three independent experiments).

We next analysed the interplay of ATP, lactate, and pyruvate in beating human iPS cell-derived cardiomyocytes. Cardiomyocytes possess a large number of mitochondria, and effective regulation of ATP production and Ca^2+^ signalling is essential for spontaneous contraction and relaxation cycles. We investigated how these cells are affected by inhibition of oxidative phosphorylation using oligomycin (F type ATPase inhibitor), carbonyl cyanide 4-trifluoromethoxyphenylhydrazone (FCCP, uncoupling agent), rotenone (Rot, complex I inhibitor), and antimycin A (AA, complex III inhibitor). First, we monitored the Ca^2+^ dynamics with a green turn-off type Ca^2+^ indicator, Inverse-pericam^17^. Interestingly, the pulse frequency was immediately increased after the combined application of oligomycin, FCCP, Rot, and AA, but eventually declined (34.7 ± 16.9 pulses/100 s at 5 min and 11.2 ± 3.6 pulses/100 s at 50 min, Fig. 4a, Supplementary Fig. S10). To understand the mechanism of transient pulse enhancement by inhibiting oxidative phosphorylation, we observed the dynamics of lactate and pyruvate. The FI of Green Lindoblum transiently increased and returned to the basal level after application of oligomycin, FCCP, Rot, and AA (114.9 ± 12.0%, Fig. 4b, Supplementary Fig. S11a,b). The FI of Green Pegassos quickly decreased and stayed at this level (76.1 ± 7.5%, Fig. 4c, Supplementary Fig. S11c,d). Because the FI of Green Lindoblum and Green Pegassos changed in opposite directions, the results suggest that effect of pH changes may be small, and inhibition of oxidative phosphorylation causes actual changes in lactate and pyruvate levels.

Inhibition of oxidative phosphorylation generally stops ATP production in mitochondria and causes accumulation of NADH^18^. Although we cannot precisely discuss the changes in mitochondrial ATP levels, transient increase in the FI of Green Lindoblum and prolonged decrease in the FI of Green Pegassos can be explained by the perturbation of balance between ATP and NADH levels. Metabolic perturbation causes dysregulation of Ca^2+^ signalling. Impairment in Ca^2+^ uptake into sarcoplasmic reticulum by Ca^2+^-ATPase (SERCA) results in Ca^2+^ overload, which is a primary cause of arrhythmia^19^. The increase of pulse frequency immediately after application may reflect this concept. Eventually, cells exhaust ATP and pulse frequency declines (Supplementary Fig. S12). This data suggest that the metabolic condition of cardiomyocytes in combination with other FP-based indicators can be monitored. Cardiotoxicity is a major side-effect of anti-cancer agents^20^; therefore, Green Lindoblum and Green Pegassos may serve as valuable tools in high-throughput drug screening for novel anti-cancer agents with minimal alterations in myocardial functions.

## Conclusion

In this report, we developed single FP-based indicators for lactate and pyruvate and revealed their utility for various applications both in vitro and in living cells. These new indicators will shed new light on monitoring or measuring metabolic molecules and even gliotransmitters and help to understand the functional relationship between cell signalling and metabolic activity.

## Methods

### Chemicals

Lactic acid and oxaloacetic acid were purchased from Tokyo Chemical Industry (Tokyo, Japan). Pyruvate, phosphoenolpyruvic acid, citric acid, pyridoxal monophosphate (PLP), forskolin, and glucose were purchased from FUJIFILM Wako Pure Chemical Corporation (Osaka, Japan). Acetyl-CoA, L-glutamine, and L-serine were purchased from Nacalai Tesque (Kyoto, Japan). D-serine, L-glutamate monosodium salt monohydrate, and sodium L-lactate were purchased from Sigma-Aldrich (St. Louis, MO, USA).

### Plasmid construction

The DNA fragment of green fluorescent protein (GFP), used in a green Ca^2+^ indicator G-GECO^21^, was synthesised by Integrated DNA Technologies (Coralville, IA, USA). This was engineered to contain *Kpn*I and *Bgl*II restriction enzyme sites between N145 and S146 and then cloned into the pRSET-A vector (Thermo Fisher Scientific, Waltham, MT, USA) at the *Xho*I/*Hind*III site. The lactate binding domain of the lactate dehydrogenase transcriptional regulator (LldR, WP_001297449.1, amino acids 86–260) from the FRET-type lactate indicator Laconic, or the DNA-binding domain, and the pyruvate-binding domain of the pyruvate dehydrogenase transcriptional regulator (PdhR, NP_414655.1) derived from the FRET-type pyruvate indicator Pyronic were both separately cloned into the *Kpn*I/*Bgl*II site of GFP^2,3^. To increase the protein solubility from expression in *Escherichia coli*, the sequence of the super-acidic region of mouse amyloid β precursor protein (APP, NM_001198823.1, amino acids 190–286) was fused to the N-terminal side of the GFP in pRSET-A^22^. To improve the fluorescence intensity change by linker length, leucine zipper sequences of various lengths were inserted between the GFP and the lactate/pyruvate-binding domain^23^. The mutant that produced the greatest change in fluorescence intensity in the absence or presence of lactate/pyruvate was selected for further optimisation. Polymerase chain reaction (PCR) was then performed using two sets of primers including NNK and MNN to introduce random mutations at one position in the linker amino acid sequence. Approximately 50 random mutants were produced, and the mutant with the maximum dynamic range was selected as the next template for random PCR. After the repetitive screening, a mutant that produced the largest change in fluorescence intensity through interaction with the lactate/pyruvate was named as Green Lindoblum or Green Pegassos, respectively. For mammalian expression, the optimised fluorescent mutants were subcloned into the pcDNA3.1(-) vector (Thermo Fisher Scientific). Green Lindoblum was subcloned with the APP super-acidic region for improved solubility in mammalian cells. To target Green Lindoblum and Green Pegassos to plasma membrane, CAAX box sequence (KMSKDGKKKKKKSKTKCVIM) was fused to the C-terminus to each indicator and subcloned into pcDNA3.1(+) vector. Confocal images were acquired to investigate their localisation using a laser confocal microscope (C2 + , Nikon, Tokyo, Japan) equipped with an oil immersion × 100 objective lens (Plan Apo VC, × 100, NA = 1.40, Nikon) and 488 nm laser (Sapphire, Coherent, Santa Clara, CA, USA). Inverse-pericam plasmid in pcDNA3 was a kind gift from Dr. Atsushi Miyawaki^17^. Pink Flamindo plasmid was used as described in Harada et al^24^. D-serine dehydratase gene from *Saccharomyces cerevisiae* (Dsd1p) in pET5b vector was a kind gift from Dr. Tomokazu Ito^13,14^.

### Mutant screening

pRSET-A with Green Lindoblum was transformed into *E. coli* BL21 (DE3) (Merck Millipore, Burlington, MA, USA) cells and cultured in 3 mL LB medium with 50 μg/mL ampicillin (FUJIFILM Wako Pure Chemical Corporation, Osaka, Japan) at 20 °C for 2 to 3 days. pRSET-A with Green Pegassos was transformed into *E. coli* JM109 (DE3) (Promega, Madison, WI, USA) cells and cultured in 200 mL LB medium with 50 μg/mL ampicillin (FUJIFILM Wako Pure Chemical Corporation) at 20 °C for 3 days. The cells were harvested by centrifugation at 16,000 g for 5 min at 4 °C. The pellets were suspended in phosphate-buffered saline (PBS; pH 7.4) with 40 μg/mL lysozyme (FUJIFILM Wako Pure Chemical Corporation) and 0.5% Triton X-100 (Sigma-Aldrich, for Green Lindoblum only), and lysed by ultrasonic homogenisation. After centrifugation at 16,000 *g* for 10 min at 4 °C of the lysate, the supernatant containing the over-expressed Green Lindoblum/Pegassos protein was added to PBS. Emission spectra of 470 nm in the absence or presence of 10 mM lactate or 1 mM pyruvate were measured using a spectro-fluorophotometer (F-2500, Hitachi, Tokyo, Japan). The dynamic range (F/F_0_) was normalised to the peak in the absence of lactate or pyruvate, and the variant showing the highest F/F_0_ was selected.

### Protein expression and purification

Experimental procedures were mainly based on our previous works^7,24^. Briefly, pRSET-A with Green Lindoblum was transformed into *E. coli* BL21 (DE3) (Merck Millipore, Burlington, MA, USA) cells and cultured in 200 mL LB medium with 50 μg/mL ampicillin (FUJIFILM Wako Pure Chemical Corporation, Osaka, Japan) at 20 °C for 2 days. pRSET-A with Green Pegassos was transformed into *E. coli* JM109 (DE3) (Promega, Madison, WI, USA) cells and cultured in 200 mL LB medium with 50 μg/mL ampicillin (FUJIFILM Wako Pure Chemical Corporation) at 20 °C for 4 days. The cells were harvested by centrifugation at 4800 *g* for 10 min at 4 °C. The pellets were suspended in phosphate-buffered saline (PBS; pH 7.4) with 40 μg/mL lysozyme (FUJIFILM Wako Pure Chemical Corporation) and 0.5% Triton X-100 (Sigma-Aldrich, for Green Lindoblum only), and lysed by three-freeze thaw cycles, followed by ultrasonic homogenisation. After centrifugation at 4800 *g* for 20 min at 4 °C of the lysate, the supernatant containing the over-expressed Green Lindoblum/Pegassos protein was recovered. The Green Lindoblum-containing lysate was stored at 4 °C until further use without purification. The Green Pegassos-containing lysate was applied to nickel-nitrilotriacetate (Ni-NTA) beads (QIAGEN, Venlo, Netherlands) for purification. After washing three times with 10 mM imidazole/PBS, the protein was eluted with 300 mM imidazole/PBS. To remove imidazole, the elution was added to a PD-10 gel filtration column (GE Healthcare, Buckinghamshire, UK) in HEPES buffer (150 mM KCl, 50 mM HEPES-KOH, pH 7.4). The purified proteins were aliquoted and stored at − 80 °C until further use.

### In vitro spectroscopy

Excitation (530 nm)/emission (470 nm for Green Lindoblum and 480 nm for Pegassos) spectrum measurements were carried out in the absence or presence of 10 mM lactate or 1 mM pyruvate using a spectro-fluorophotometer (F-2500, Hitachi, Tokyo, Japan). To generate a dose-response curve, the fluorescence intensity of the purified proteins was measured in the presence of various concentrations of lactate/pyruvate solution (0–10 mM). Excitation/emission spectra and dose-response curves were measured in PBS for Green Lindoblum and HEPES buffer for Green Pegassos. The EC_50_ value was calculated using the Rodbard mode in the Curve Fitter function in ImageJ (National Institutes of Health, Bethesda, MD, USA) using a four-parameter logistic curve equation where *b* and *c* corresponds to Hill coefficient and EC_50_, respectively:

$$\mathrm{y}=d+\frac{a-d}{1+{\left(\frac{x}{c}\right)}^{b}}$$

To obtain pH-dependent data, purified Green Lindoblum/Pegassos proteins in buffers (for Green Lindoblum, 150 mM KCl, 50 mM HEPES-KOH, pH 5.0–7.0; 150 mM KCl, 50 mM Tris-KOH in pH 7.5–10.0; for Green Pegassos, 150 mM KCl, 100 mM HEPES-KOH, pH 5.0–8.0; 150 mM KCl, 100 mM Tris-KOH, pH 8.5–9.0) with various pHs were measured in the absence or presence of 10 mM lactate or 1 mM pyruvate. To examine the ligand specificity, the fluorescence intensity was measured of Green Lindoblum/Pegassos with various molecules (for Green Lindoblum, 3 mM oxaloacetic acid, pyruvate, phosphoenolpyruvic acid, citric acid, glucose, D-serine, L-serine, L-glutamine, and L-glutamate; for Green Pegassos, 700 μM oxaloacetic acid, acetyl-CoA, citric acid, phosphoenolpyruvic acid, lactate, D-serine, L-serine, L-glutamine, and L-glutamate).

### Blood collection from mice and plasma lactate measurement

All experiments with mice were approved by the Animal Experiment Ethics Committee of the Graduate School of Arts and Sciences in The University of Tokyo (approval no. 29-4). Experiments were performed by the guidelines by Life Science Research Ethics and Safety Committee of The University of Tokyo. Slc:ICR mice were purchased from Japan SLC (Shizuoka, Japan) at 7 to 30 weeks-old and fasted overnight with access to water. Mice were anaesthetised with isoflurane, and blood was collected from the inferior vena cava with 1 mg/mL of EDTA. For lactate tolerance test, mice were kept on free access to both food and water, and blood was collected from the tail vein before and 10 min after intraperitoneal injection of 1 g/kg sodium L-lactate (20% w/v solution in saline). Blood was centrifuged at 1000 *g* for 5–10 min at 4 °C, and 5 μL of plasma was added to PBS containing Green Lindoblum protein for a final volume of 500 μL. Fluorescence intensity was measured in the same manner as in the dose-response curve, and the lactate level was calculated from a four-parameter logistic curve equation. Simultaneously, the blood lactate level was measured with a commercial lactate test meter (Lactate Pro 2, Arkray, Kyoto, Japan). Correlation between the level calculated from Green Lindoblum and the level measured by Lactate Pro 2 was investigated using Pearson’s product-moment correlation in R.

### D-serine measurement from mouse primary cultured neurons and astrocytes

D-serine dehydratase gene from *Saccharomyces cerevisiae* (Dsd1p) was subcloned into *Xho*I/*Hind*III sites of pRSET-A vector and fused with the super-acidic region of APP at the N-terminus. Dsd1p protein was harvested and purified in the same manner as Green Pegassos. To obtain a dose-response to D-serine, known concentrations of D-serine, PLP (20 μM), and Dsd1p protein (2 μg) were mixed in artificial cerebrospinal fluid (aCSF, 125 mM NaCl, 3 mM KCl, 24 mM NaHCO_3_, 1.25 mM KH_2_PO_4_, 1 mM CaCl_2_, 1.25 mM MgSO_4_, 30 mM glucose, pH 7.4^25^) and incubated for 30 min at 37 °C. Then 1 μM of Green Pegassos protein was added and incubated for 20 min at 37 °C, and the fluorescence intensity was measured. The EC_50_ value was calculated as in the dose-response curve to pyruvate. For the collection of mouse primary neurons and astrocytes, pregnant Slc:ICR mouse was purchased from Japan SLC, and pups at P0 were anaesthetised using isoflurane; the brain was collected, and olfactory bulb and cerebellum were removed. Tissues were minced in washing medium (Dulbecco’s modified Eagle medium, DMEM [Sigma-Aldrich]: Hank’s balanced salt solution, HBSS [Thermo Fisher Scientific] at a 2:1 ratio supplemented with 42.5 mM glucose, 29.3 mM NaHCO_3_, 31.5 mM HEPES, pH 7.2) and suspended in 5 mL of enzyme solution (PBS: HBSS, at ratio of 4:1, with 9 U/mL papain [Worthington, Columbus, OH, USA], 0.16 mM L-cysteine, 20 μg/mL BSA, 0.5 mg/mL glucose, 0.1 mg/mL DNase I [Sigma-Aldrich], 1.2 mM MgSO_4_) for 15 min at 37 °C. Then, 1 mL of culture medium (Neurobasal medium, Thermo Fisher Scientific) supplemented with 10% horse serum (Thermo Fisher Scientific), 2 mM L-glutamine (Thermo Fisher Scientific), 10 μg/mL gentamycin (Thermo Fisher Scientific), and B27 (Thermo Fisher Scientific) was added and centrifuged at 300 g for 5 min at room temperature. The pellet was suspended in a culture medium, and cells were seeded in 6 well plates at 2 × 10^5^ cells per well. The medium was exchanged every 3 to 5 days with high glucose DMEM supplemented with glutamine, sodium pyruvate, 10% (v/v) foetal bovine serum (FBS, Sigma-Aldrich), 100 U/mL penicillin, and 0.1 mg/mL streptomycin (Sigma-Aldrich). Fifteen days after plating, cells were washed twice with aCSF and incubated in 1 mL of control aCSF or aCSF containing 300 μM allyl isothiocyanate (AITC, FUJIFILM Wako Pure Chemical Corporation) or 1 mM serotonin (FUJIFILM Wako Pure Chemical Corporation) at 37 °C with 5% CO_2_. After centrifugation at 1000 g for 10 min at 4 °C, supernatant was mixed with Dsd1p, PLP, and Green Pegassos as described above. Measured fluorescence intensity was fitted with the dose-response curve for D-serine to estimate the D-serine level. Samples that showed lower fluorescence intensity than standard 1 μM D-serine were defined as “not detected” (ND).

### Cell culture and transfection

HEK293T and HeLa cells were cultured in high glucose DMEM supplemented with L-glutamine, sodium pyruvate, 10% (v/v) FBS, 100 U/mL penicillin, and 0.1 mg/mL streptomycin. HeLa cells (0.5 × 10^5^) or HEK293T cells (0.45 × 10^5^) were seeded in 35 mm glass-bottomed dishes coated with 1 mg/mL poly-L-lysine (Sigma-Aldrich) and cultured for 2 days. Green Lindoblum/Pegassos expression plasmids (1.5 μg) were transfected using 3 μL of Lipofectamine 2000 Transfection Reagent (Thermo Fisher Scientific) in 1 mL of medium. Cells were incubated at 37 °C for 4 h, and after changing the medium cells were cultured at 32 °C and 37 °C for 2 days until imaging.

Human iPS cell-derived cardiomyocytes (CarmyA, Myoridge, Kyoto, Japan) were initiated in 24 well plates with seeding medium (#ME-02, Myoridge) supplemented with 10 μM Y-27632 (Culture Sure, FUJIFILM Wako Pure Chemical Corporation) and 0.5 μg/mL laminin (iMatrix-511 silk, nippi, Tokyo, Japan). The medium was exchanged every day with maintaining medium (#ME-01, Myoridge). About 1 week after plating, cells were washed with PBS and detached using Accumax (Nacalai Tesque). After centrifugation at 300 g for 5 min at room temperature, 4.5 × 10^4^ cells were seeded in multi-well 35 mm glass-bottomed dishes as described in initiation. Medium was exchanged every day with maintaining medium, and 2 days after plating, 0.5–0.8 μg of plasmids were transfected using 1.5–2.4 μL of ViaFect Transfection Reagent (Promega) in 400 μL of maintaining medium. Cells were incubated at 37 °C for 4 h, and after medium changing, the cells were cultured at 37 °C for 2 days until imaging.

### Live cell imaging

Experimental procedures were mainly based on our previous works^7,24^. Briefly, cells were incubated with modified Ringer’s buffer (RB: 140 mM NaCl, 3.5 mM KCl, 0.5 mM NaH_2_PO_4_, 0.5 mM MgSO_4_, 1.5 mM CaCl_2_, 10 mM HEPES, 2 mM NaHCO_3_) for 30 min at 37 °C with 5% CO_2_ and subsequently used for imaging. For dual-colour imaging with HeLa cells, cells were loaded with 1 μM of Rhod2 (Abcam, Cambridge, UK) for 30 min during incubation and washed twice with RB. Single-colour imaging of HEK293T and HeLa cells was performed using a fluorescence inverted microscope (IX-71, Olympus, Tokyo, Japan) equipped with an oil immersion × 40 objective lens (UApo/340, × 40, NA = 1.35, Olympus), an EM-CCD camera (Evolve, Photometrics, Tucson, AZ, USA), and a xenon lamp. Images were acquired using a filter set (U-MWIBA2, Olympus) of an excitation filter of 460–495 nm, an emission filter of 510–550 nm, and a dichroic mirror of 505 nm. For FRET imaging, split imaging port (U-SIP, Olympus) and a filter set (CYP/FYP split view, Olympus) of an excitation filter of 436 nm, a dual-band emission filter of 460 and 525 nm, and a dichroic mirror of 455 nm were used. Dual-colour imaging in HeLa cells and human iPS cell-derived cardiomyocytes was performed using a fluorescence inverted microscope (Axio Observer D1, Carl Zeiss, Oberkochen, Germany) equipped with an oil immersion × 40 objective lens (UApo/340, × 40, NA = 1.35, Olympus) and a CMOS camera (ORCA-Flash4.0V2, C11440, Hamamatsu Photonics, Shizuoka, Japan). Fluorescence images were acquired using a mercury lamp (HBO 100, Carl Zeiss), with a BP470/40 and FT495 filter (B38-HE, Carl Zeiss) for Green Lindoblum/Pegassos, and BP530-585 and FT600 (G00, Carl Zeiss) for Rhod2. The lens, stage, and perfusion tube were heated to 37 °C. Images were acquired every 5 s using MetaMorph software (Molecular Devices, Sunnyvale, Calif., USA).

### Imaging with human iPS cell-derived cardiomyocytes

Cells on multi-well glass-bottomed dishes were washed twice and observed in HEPES-Tyrode solution (140 mM NaCl, 2.7 mM KCl, 1.8 mM CaCl_2_, 1.2 mM NaHCO_3_, 0.49 mM MgCl_2_, 0.37 mM NaH_2_PO_4_, 25 mM HEPES, 5 mM glucose, pH 7.4). Imaging was performed with the same equipment used in dual-colour imaging as described above. For imaging with Inverse-pericam, cells were imaged for 100 s at 20 frames per second every 5 min; 1 μM oligomycin, 1 μM carbonyl cyanide 4-trifluoromethoxyphenylhydrazone (FCCP), and 250 nM rotenone/antimycin A (Agilent, Santa Clara, CA, USA) were applied in 40 μL of HEPES-Tyrode buffer immediately after the initial image acquisition was finished. For imaging with Green Lindoblum, and Green Pegassos, cells were imaged every 5 s for 55 min. Oligomycin and FCCP and rotenone/antimycin A were applied 5 min after the beginning of image acquisition.

### Imaging data and statistical analysis

Dose-response curves were fitted with four-parameter logistic curves. The X–Y drift of imaging data was corrected using the ImageJ plug-in, Stackreg, and the fluorescence intensity of each cell was measured using MetaMorph software. For imaging with HeLa, HEK293T, and GLUTag cells, average fluorescence intensity during 30 s before stimulation was normalised to 100%. Cells that exhibited photobleaching of more than 20% before stimulation were removed from data analysis. For imaging with human iPS cell-derived iPS cells, average fluorescence intensity during 5 min before stimulation was normalised to 100%. To count the pulse frequency of cardiomyocytes, we measured the fluorescence intensity of Inverse-pericam and counted the number of dimming spikes. Data are shown as means ± standard deviation. Statistical analysis was performed with GraphPad Prism software (GraphPad Software, San Diego, CA, USA). Means were compared by Welch’s t test or unpaired *t* test.

## Supplementary information

Supplementary Figures.
