# Supplementary material for: Green fluorescent protein-based lactate and pyruvate indicators suitable for biochemical assays and live cell imaging
Source: Sci Rep. 2020 Nov 11;10:19562. doi: 10.1038/s41598-020-76440-4 (PMC7659002; doi:10.1038/s41598-020-76440-4)
Supplement: Supplementary file 1 — Supplementary Figures. [file 41598_2020_76440_MOESM1_ESM.docx]

**Online Supplementary Information**

**Green Fluorescent Protein-Based Lactate and Pyruvate Indicators Suitable for Biochemical Assays and Live Cell Imaging**

Kazuki Harada^1, 7^, Takami Chihara^2, 7^, Yuki Hayasaka^1, 7^, Marie Mita^1^, Mai Takizawa^1^, Kentaro Ishida^3^, Mary Arai^4^, Saki Tsuno^5^, Mitsuharu Matsumoto^5^, Takeshi Ishihara^4^, Hiroshi Ueda^6^, Tetsuya Kitaguchi^6, *^ and Takashi Tsuboi^1, 2, *^

^1^ Department of Life Sciences, Graduate School of Arts and Sciences, The University of Tokyo, 3-8-1 Komaba, Meguro, Tokyo 153-8902, Japan

^2^ Department of Biological Sciences, Graduate School of Science, The University of Tokyo, 7-3-1 Hongo, Bunkyo, Tokyo 113-0033, Japan

^3^ Myoridge Co. Ltd., 46-29 Yoshidashimoadachi-cho, Sakyo, Kyoto 606-8501, Japan

^4^ Department of Biology, Faculty of Science, Kyushu University, 774 Motooka, Nishi, Fukuoka 819-0395, Japan

^5^ Dairy Science and Technology Institute, Kyodo Milk Industry Co. Ltd., Hinode-machi, Nishitama-gun, Tokyo 190-0182, Japan

^6^ Laboratory for Chemistry and Life Science, Institute of Innovative Research, Tokyo Institute of Technology, 4259 Nagatsuta-cho, Midori-ku, Yokohama, Kanagawa, 226-8503, Japan

^*^ Tetsuya Kitaguchi, Ph.D. E-mail: kitaguct-gfp@umin.ac.jp

^*^ Takashi Tsuboi, Ph.D. E-mail: takatsuboi@bio.c.u-tokyo.ac.jp

^7^ These authors contributed equally.

**Supplementary Figure S1. Diagrams for describing the screening processes of Green Lindoblum.** (**a, b**) Schematic drawing of the prototype indicator and various candidates for the linker in the C- (**a**) and N- (**b**) termini. Prototype 1 contains the DNA-binding domain and the ligand binding domain of LldR, whereas Prototype 2 contains only the ligand binding domain. Fluorescence responses to 10 mM lactate were compared and the highest response (Prototype 2, C + 12, and N − 6) was used as the template of point mutations. (**c**) Processes of point mutations into the candidate with optimal linker lengths (Prototype 2, N – 6, and C + 12). Asterisks represent introduced mutations. Dynamic ranges to 10 mM lactate (F/F_0_) were compared and the resultant construct with the highest F/F_0_ was named Green Lindoblum.


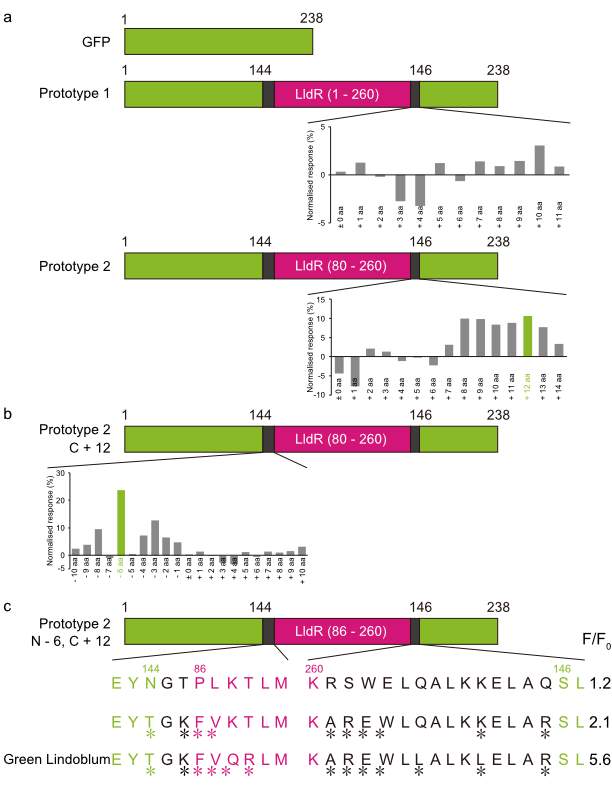

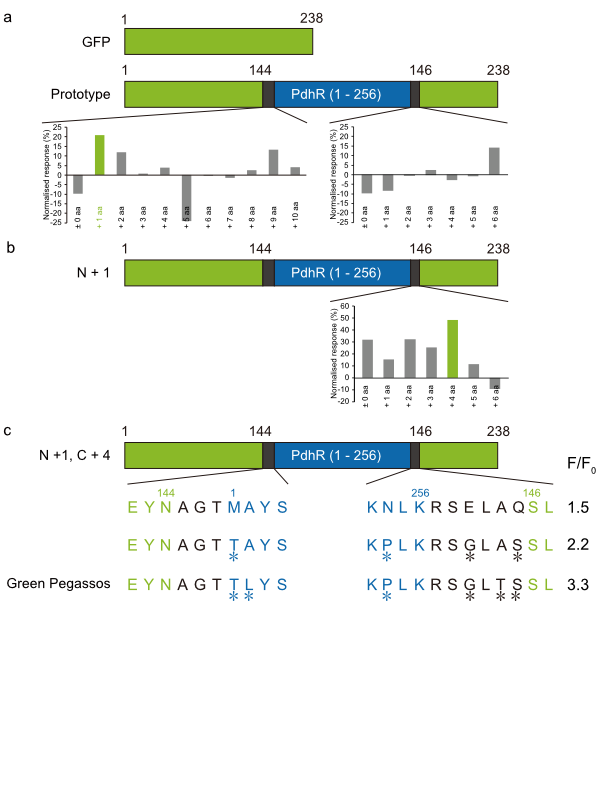


**Supplementary Figure S2. Diagrams for describing the screening processes of Green Pegassos.** (**a, b**) Schematic drawing of the prototype indicator and various candidates for the linker in the N- (**a**) and C- (**b**) termini. Fluorescence responses to 1 mM pyruvate were compared, and the highest response (N +1, C + 4) was used as the template of point mutations. (**c**) Processes of point mutations into the candidate with optimal linker lengths (N + 1, C + 4). Asterisks represent introduced mutations. Dynamic ranges to 1 mM pyruvate (F/F_0_) were compared, and the resultant construct with the highest F/F_0_ was named Green Pegassos.

**Green Lindoblum**

VSKGEELFTGVVPIQVELDGDVNGHKFSVSGEGEGDATYGKLTLKFICTTGKLPVPWPTL

VTTLTYGVQCFSRYPDHMKQHDFFKSAMPEGYIQERTIFFKDDGNYKTRAEVKFEGDTLV

NRIELKGIDFKEDGNILGHKLEYTGKFVQRLMADDPDYSFDILEARYAIEASTAWHAAMR

ATPGDKEKIQLCFEATLSEDPDIASQADVRFHLAIAEASHNIVLLQTMRGFFDVLQSSVK

HSRQRMYLVPPVFSQLTEQHQAVIDAIFAGDADGARKAMMAHLSFVHTTMKRFDEDQARH

ARITRLPAEHNEHSREKNALKAREWLLALKLELARSLENVYIKADKQKNGIKANFKIRHN

IEDGGVQLAYHYQQNTPIGDGPVLLPDNHYLSVQSILSKDPNEKRDHMVLLEFVTAAGIT

LGMDELYK

**Green Pegassos**

VSKGEELFTGVVPIQVELDGDVNGHKFSVSGEGEGDATYGKLTLKFICTTGKLPVPWPTL

VTTLTYGVQCFSRYPDHMKQHDFFKSAMPEGYIQERTIFFKDDGNYKTRAEVKFEGDTLV

NRIELKGIDFKEDGNILGHKLEYNAGTTLYSKIRQPKLSDVIEQQLEFLILEGTLRPGEK

LPPERELAKQFDVSRPSLREAIQRLEAKGLLLRRQGGGTFVQSSLWQSFSDPLVELLSDH

PESQYDLLETRHALEGIAAYYAALRSTDEDKERIRELHHAIELAQQSGDLDAESNAVLQY

QIAVTEAAHNVVLLHLLRCMEPMLAQNVRQNFELLYSRREMLPLVSSHRTRIFEAIMAGK

PEEAREASHRHLAFIEEILLDRSREESRRERSLRRLEQRKPLKRSGLTSSLENVYIKADK

QKNGIKANFKIRHNIEDGGVQLAYHYQQNTPIGDGPVLLPDNHYLSVQSILSKDPNEKRD

HMVLLEFVTAAGITLGMDELYK

**Supplementary Figure S3. Amino acid sequences of Green Lindoblum and Green Pegassos.** The colours of the text correspond to those shown in Supplementary Figure S1 and S2.


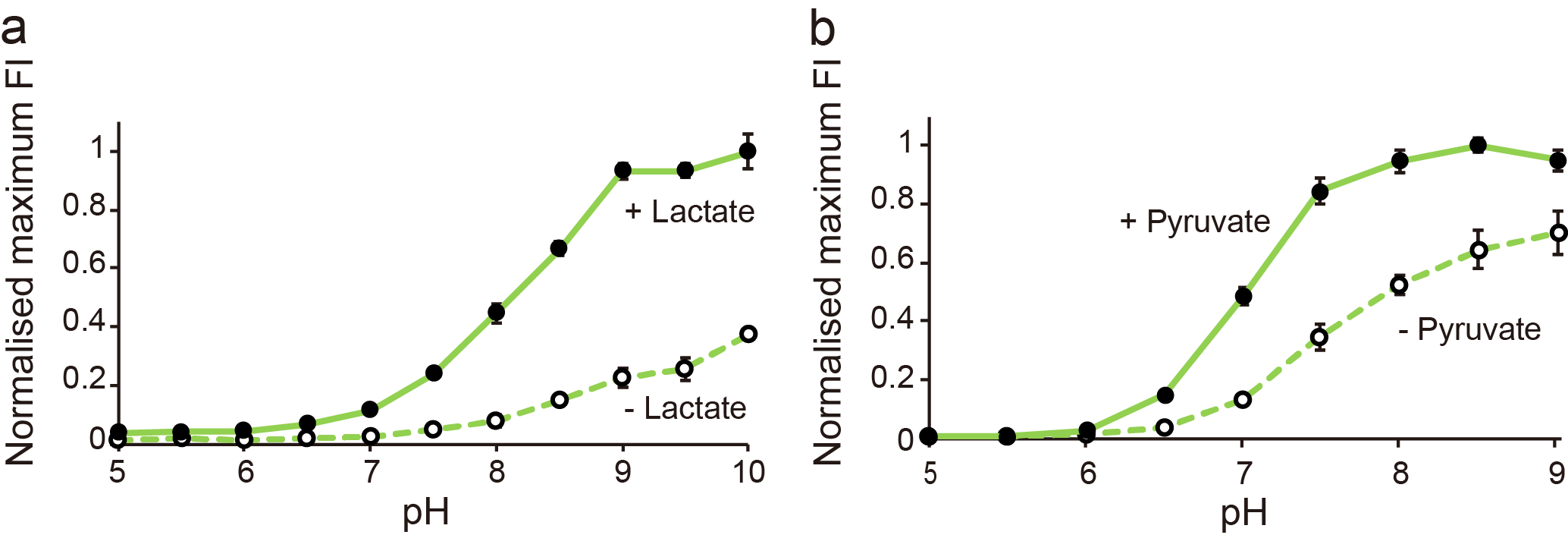


**Supplementary Figure S4. pH sensitivity of Green Lindoblum and Green Pegassos.** pH titration curves of Green Lindoblum (**a**) and Green Pegassos (**b**) in the presence (●, solid line) and absence (○, dashed line) of lactate and pyruvate, respectively. The fluorescence intensity (FI) was shown as the ratio to the peak at the highest pH in the presence of 10 mM lactate or 1 mM pyruvate. The data are shown as means ± standard deviation (n = 3).

**
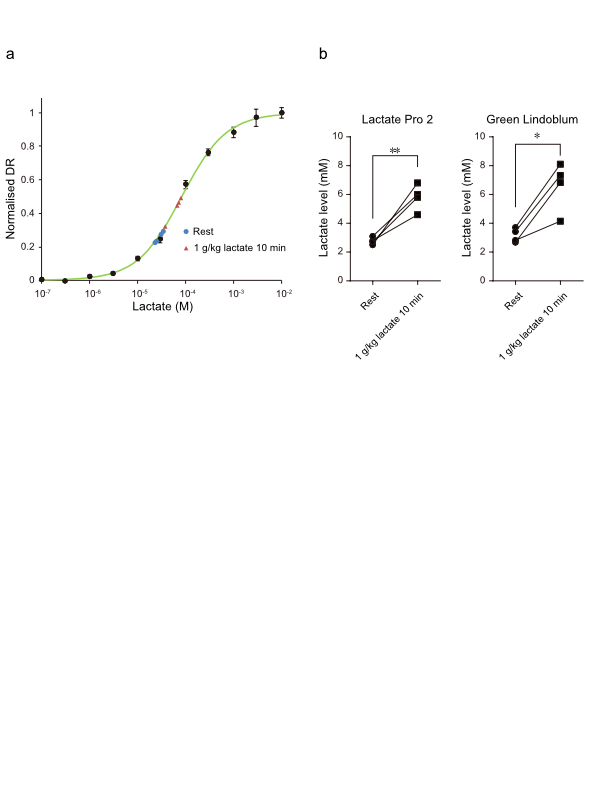
**

**Supplementary Figure S5. Measurement of plasma lactate levels in mice after lactate tolerance test using Green Lindoblum.** (**a**) Calibration of plasma lactate levels based on the dose-response curve of Green Lindoblum. The fluorescence intensity (FI) of Green Lindoblum with a 100-fold dilution of mouse plasma was plotted (cyan-filled circles and red triangles) to calculate the lactate level. The dose-response curve data are shown as means ± standard deviation (n = 3). DR, dynamic range. (**b**) Comparison of lactate levels in each mouse using Lactate Pro 2 (left) or Green Lindoblum (right) between the resting state and 10 minutes after intraperitoneal injection of 1 g/kg lactate. Student’s paired *t* test. **, *P* < 0.01; ***, *P* < 0.001.

**
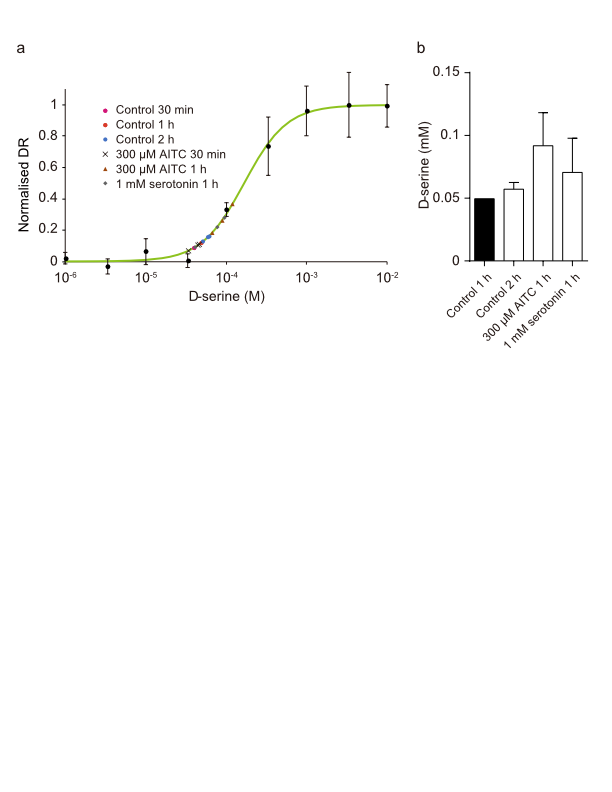
Supplementary Figure S6. Calibration of D-serine levels released from mouse primary co-cultured neurons and astrocytes by Green Pegassos.** (**a**) Dose-response curve for D-serine was made by the fluorescence intensity (FI) of Green Pegassos with known concentrations of D-serine, and the FI of supernatant from mouse primary co-cultured neurons and astrocytes were plotted. The dose-response curve data are shown as means ± standard deviation (n = 3). DR, dynamic range. (**b**) Comparison between the estimated D-serine levels from samples of Control 1 h, Control 2 h, 300 µM AITC 1 h, and 1 mM serotonin 1 h. The data are shown as means ± standard deviation (n = 1 for Control 1h, and 3 for the others). There was no significant difference between any groups.

**
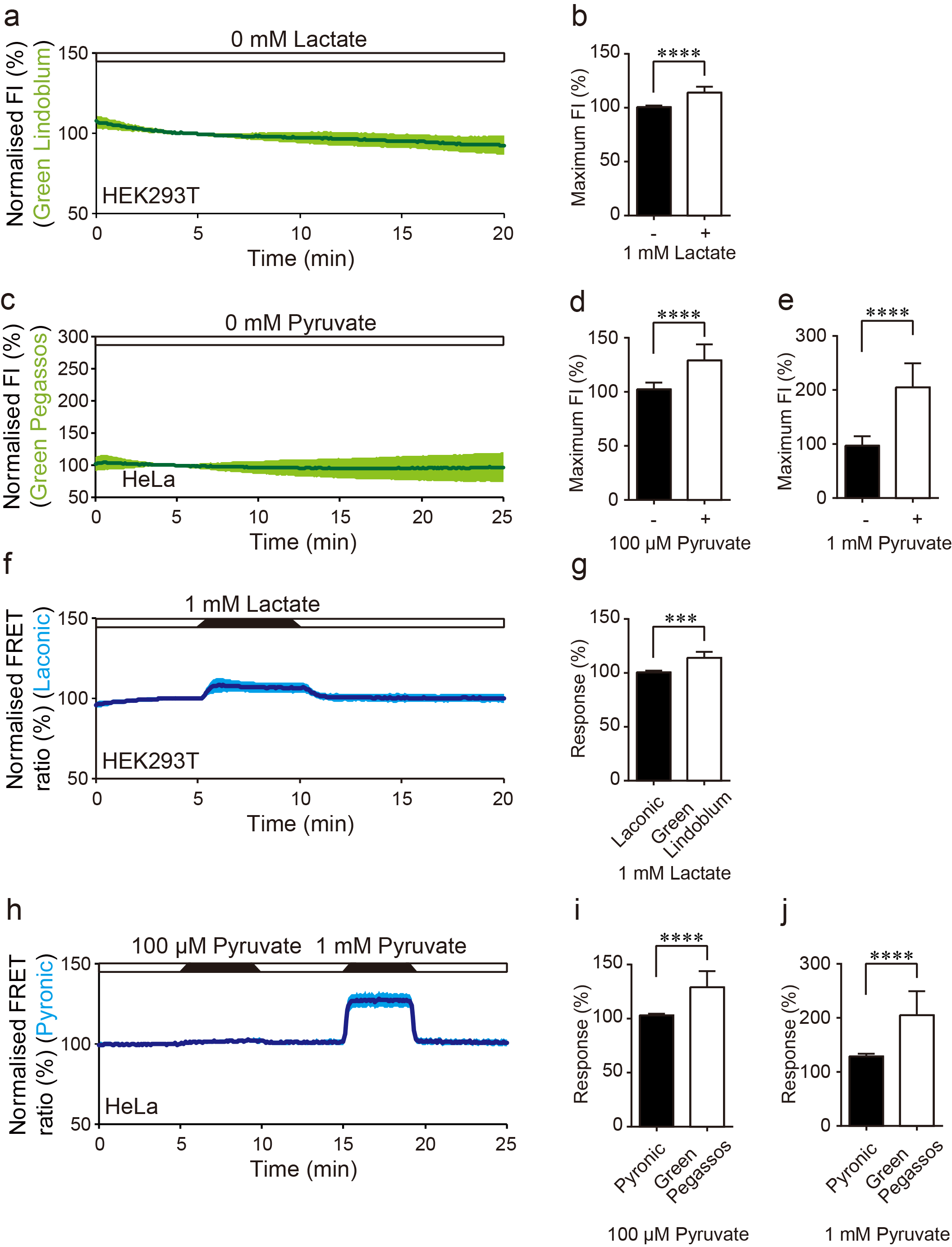
**

**Supplementary Figure S7. Live cell imaging using Green Lindoblum and Green Pegassos without stimulation, and comparison with previous FRET-based indicators Laconic and Pyronic.** (**a**) Time course of the fluorescence intensity (FI) of Green Lindoblum-expressing HEK293T cells without stimulation. (**b**) Comparison of maximum FI during 5 to 10 minutes after the beginning of image acquisition between in the presence (Fig. 3a) and the absence (Fig. S7a) of 1 mM lactate. (**c**) Time course of the fluorescence intensity (FI) of Green Pegassos-expressing HeLa cells without stimulation. (**d, e**) Comparison of maximum FI during 5 to 10 minutes (**d**) and 15 to 19 minutes (**e**) after the beginning of image acquisition between in the presence (Fig. 3b) and the absence (Fig. S7c) of 100 µM (**d**) or 1 mM (**e**) pyruvate. (**f**) Time course of the FRET ratio of Laconic-expressing HEK293T cells during the application of 1 mM lactate. (**g**) Comparison between the maximum FRET ratio of Laconic and the maximum FI of Green Lindoblum during 5 to 10 minutes in the presence of 1 mM lactate. (**h**) Time course of the FRET ratio of Pyronic-expressing HeLa cells during the application of 100 µM and 1 mM pyruvate. (**i, j**) Comparison between the maximum FRET ratio of Pyronic and the maximum FI of Green Pegassos during 5 to 10 minutes (**i**) and 15 to 19 minutes (**j**) after the beginning of image acquisition in the presence of 100 µM (**i**) or 1 mM (**j**) pyruvate. The data are shown as means ± standard deviation (n = 30 (**a**), 24 (**c**), 16 (**f**), and 14 (**h**) cells from three independent experiments). Welch’s *t* test. ***, *P* < 0.001; ****, *P* < 0.0001.


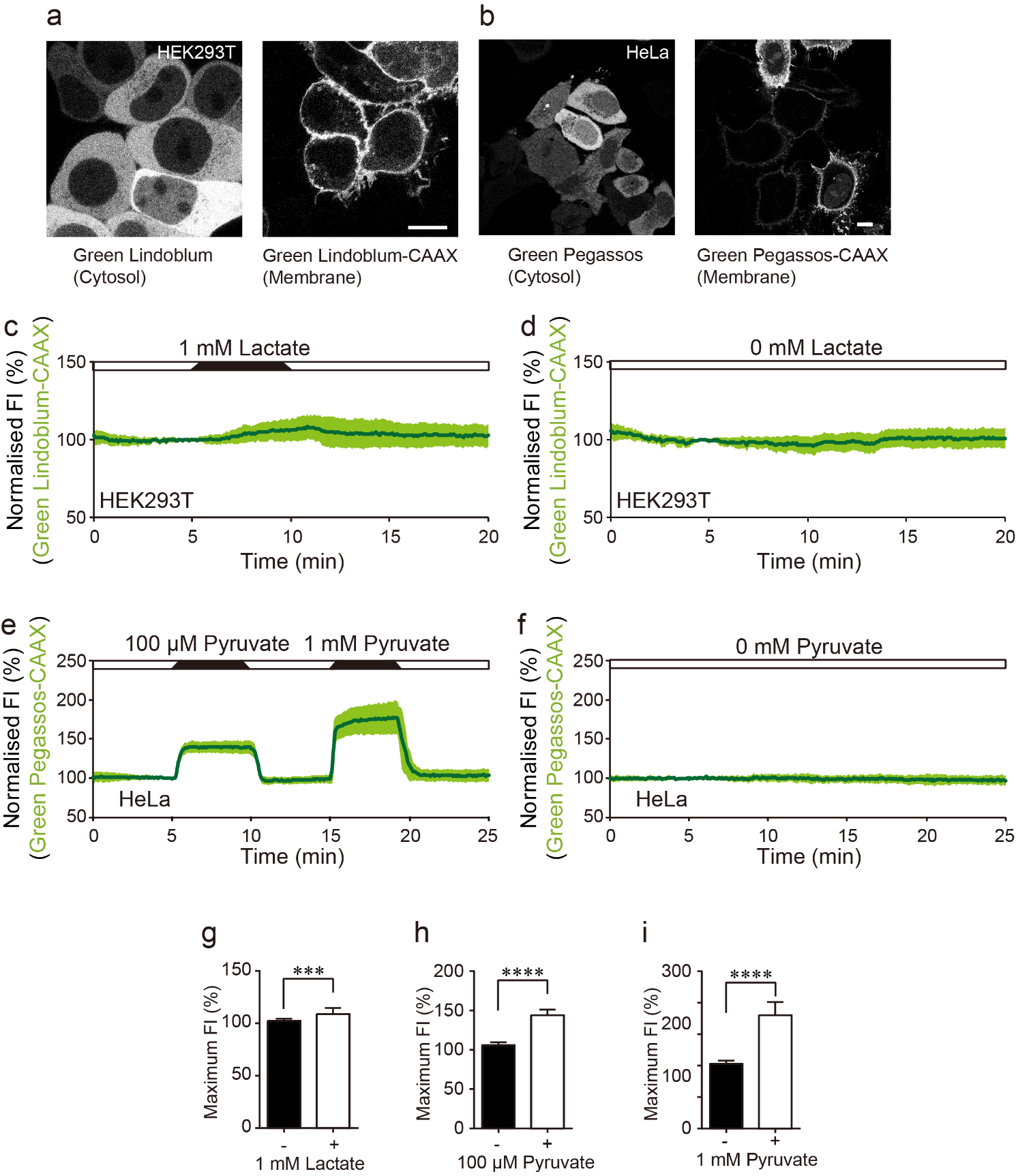


**Supplementary Figure S8. Imaging with plasma membrane-targeted Green Lindoblum and Green Pegassos.** (**a, b**) Confocal images of HEK293T cells (**a**) expressing Green Lindoblum (left) or Green Lindoblum-CAAX (right), and HeLa cells (**b**) expressing Green Pegassos (left) or Green Pegassos-CAAX (right). Scale bar represents 10 µm. (**c, d**) Time course of the fluorescence intensity (FI) of Green Lindoblum-CAAX-expressing HEK293T cells in the presence (**c**) and absence (**d**) of 1 mM lactate. (**d**) Comparison between the maximum FI of Green Lindoblum with or without CAAX during 5 to 10 minutes in the presence of 1 mM lactate. (**e, f**) Time course of the FI of Green Pegassos-CAAX-expressing HeLa cells in the presence (**e**) and absence (**f**) of 100 µM and 1 mM pyruvate. (**g**) Comparison of maximum FI of Green Lindoblum-CAAX during 5 to 10 minutes after the beginning of image acquisition between in the presence and the absence of 1 mM lactate. (**h, i**) Comparison of maximum FI during 5 to 10 minutes (**h**) and 15 to 19 minutes (**i**) after the beginning of image acquisition between in the presence and the absence of 100 µM (**h**) or 1 mM (**i**) pyruvate. The data are shown as means ± standard deviation (n = 15 (**c**, **e**) cells from three independent experiments). Welch’s *t* test. ***, *P* < 0.001; ****, *P* < 0.0001.


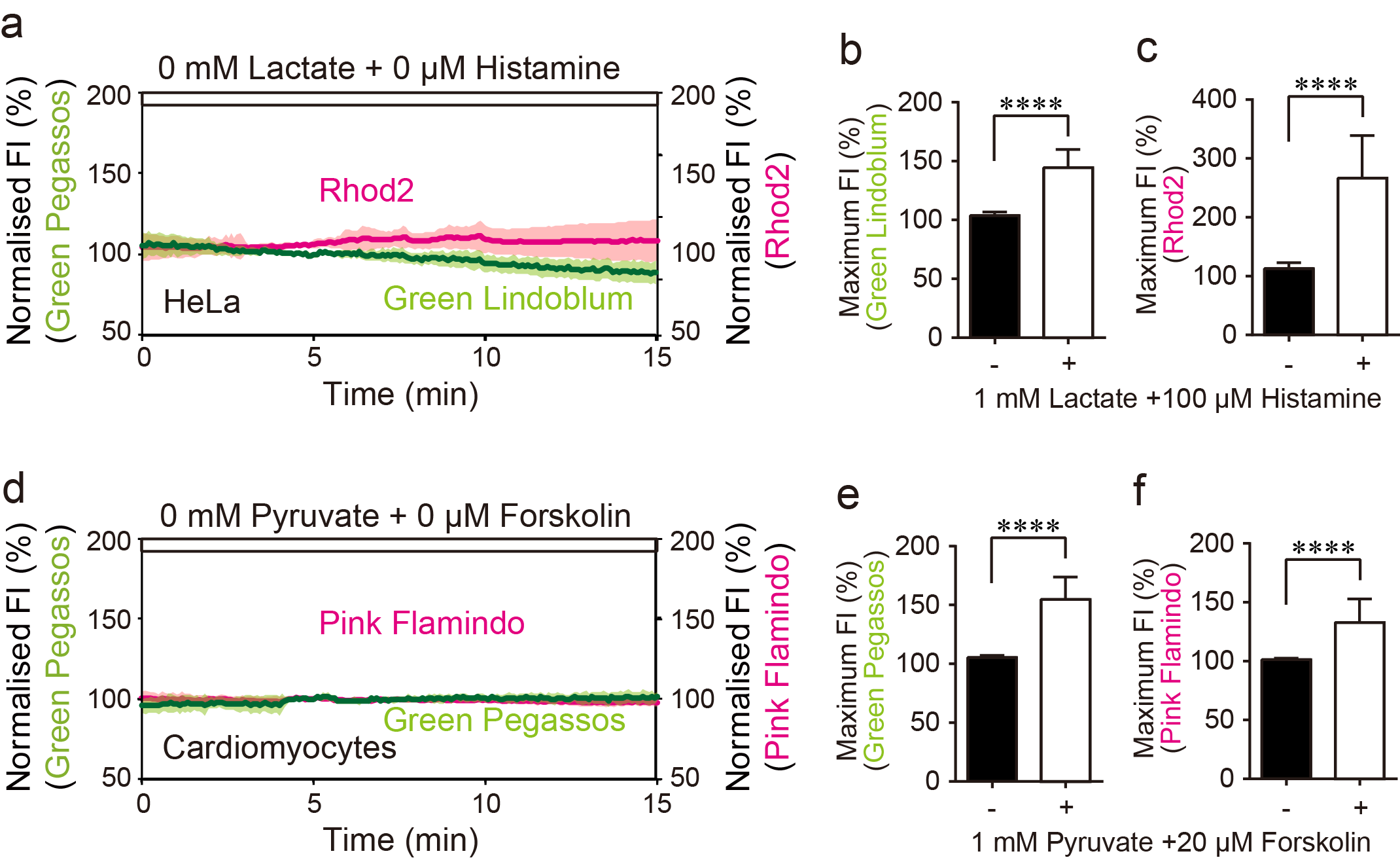


**Supplementary Figure S9. Dual-colour imaging using Green Lindoblum and Green Pegassos without stimulation.** (**a**) Time courses of the fluorescence intensity (FI) of Green Lindoblum and Rhod2 in HeLa cells without stimulation. (**b, c**) Comparison of maximum FI in Green Lindoblum (**b**) and Rhod2 (**c**) in the presence (Fig. 3c) and the absence (Fig. S9a) of 1 mM lactate plus 100 µM histamine. (**d**) Time courses of the fluorescence intensity (FI) of Green Pegassos and Pink Flamindo in human iPS cell-derived cardiomyocytes without stimulation. (**e, f**) Comparison of maximum FI in Green Pegassos (**e**) and Pink Flamindo (**f**) in the presence (Fig. 3d) and the absence (Fig. S9d) of 1 mM pyruvate plus 20 µM forskolin. The data are shown as means ± standard deviation (n = 25 (**a**) and 13 (**d**) cells from three independent experiments). Welch’s *t* test. ****, *P* < 0.0001.


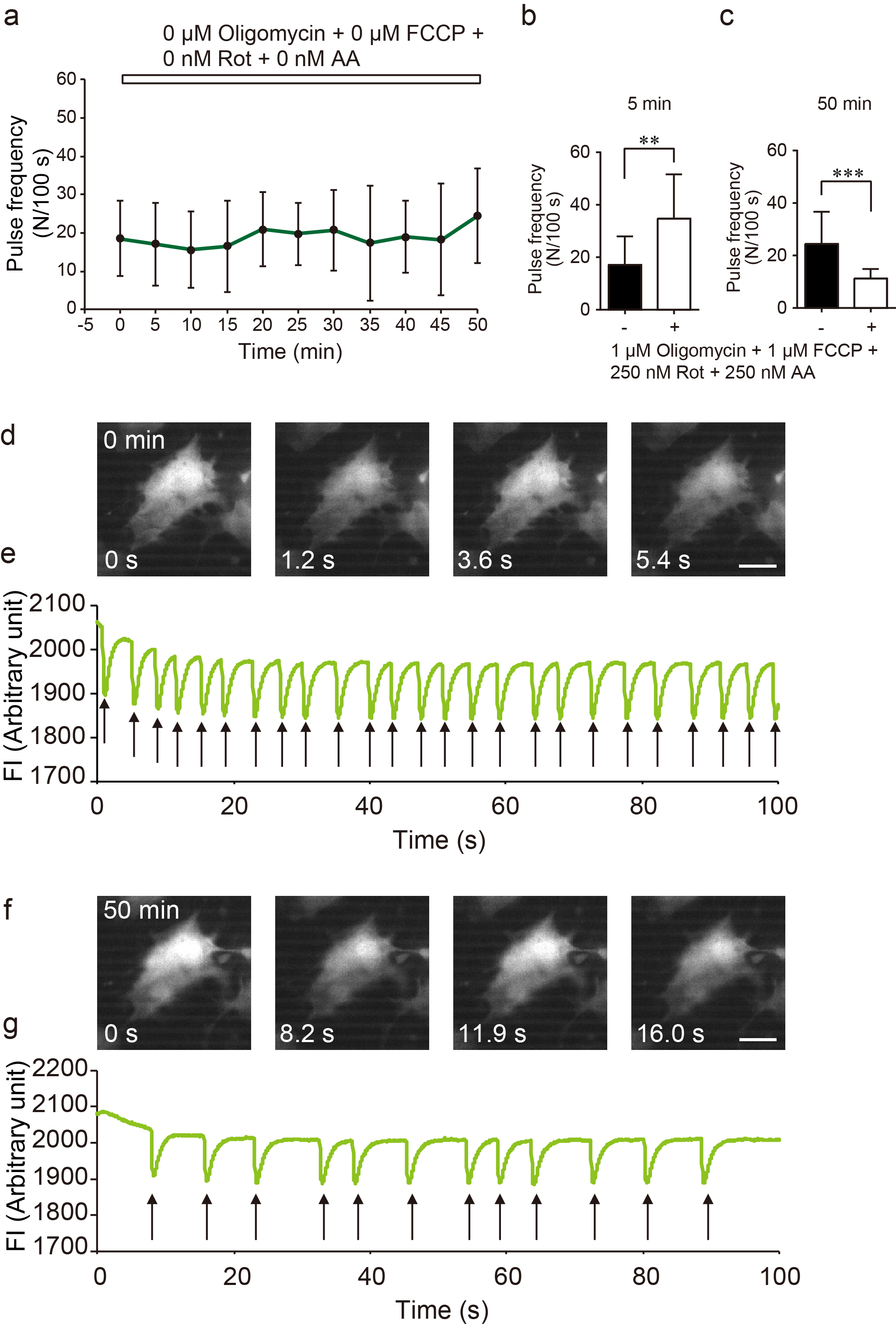


**Supplementary Figure S10. Pulse frequency without inhibition and sequential images of human iPS cell-derived cardiomyocytes expressing Inverse-pericam.** (**a**) Pulse frequency without inhibitors (n = 16 cells from three independent experiments). (**b, c**) Comparison of pulse frequency in the absence (Fig. S10a) and presence (Fig. 4a) of 1 μM oligomycin, 1 μM FCCP, 250 nM Rot, and 250 nM AA 5minutes (**b**) and 50 minutes (**c**) after application. (**d**) Typical sequential images of a human iPS cell-derived cardiomyocyte expressing Inverse-pericam at the basal state. Scale bar represents 30 µm. (**e**) Time course of the fluorescence intensity (FI) of Inverse-pericam in the cell shown in **d**. (**f**) Sequential images of the same cell 50 minutes after application of 1 μM oligomycin, 1 μM FCCP, 250 nM Rot, and 250 nM AA. Scale bar represents 30 µm. (**g**) Time course of the fluorescence intensity (FI) of Inverse-pericam in the cell shown in **f**. Arrows indicate the point of dimming spikes, which corresponds with the peak of intracellular Ca^2+^ level and the timing of contraction. Welch’s *t* test. **, *P* < 0.01; ***, *P* < 0.001.
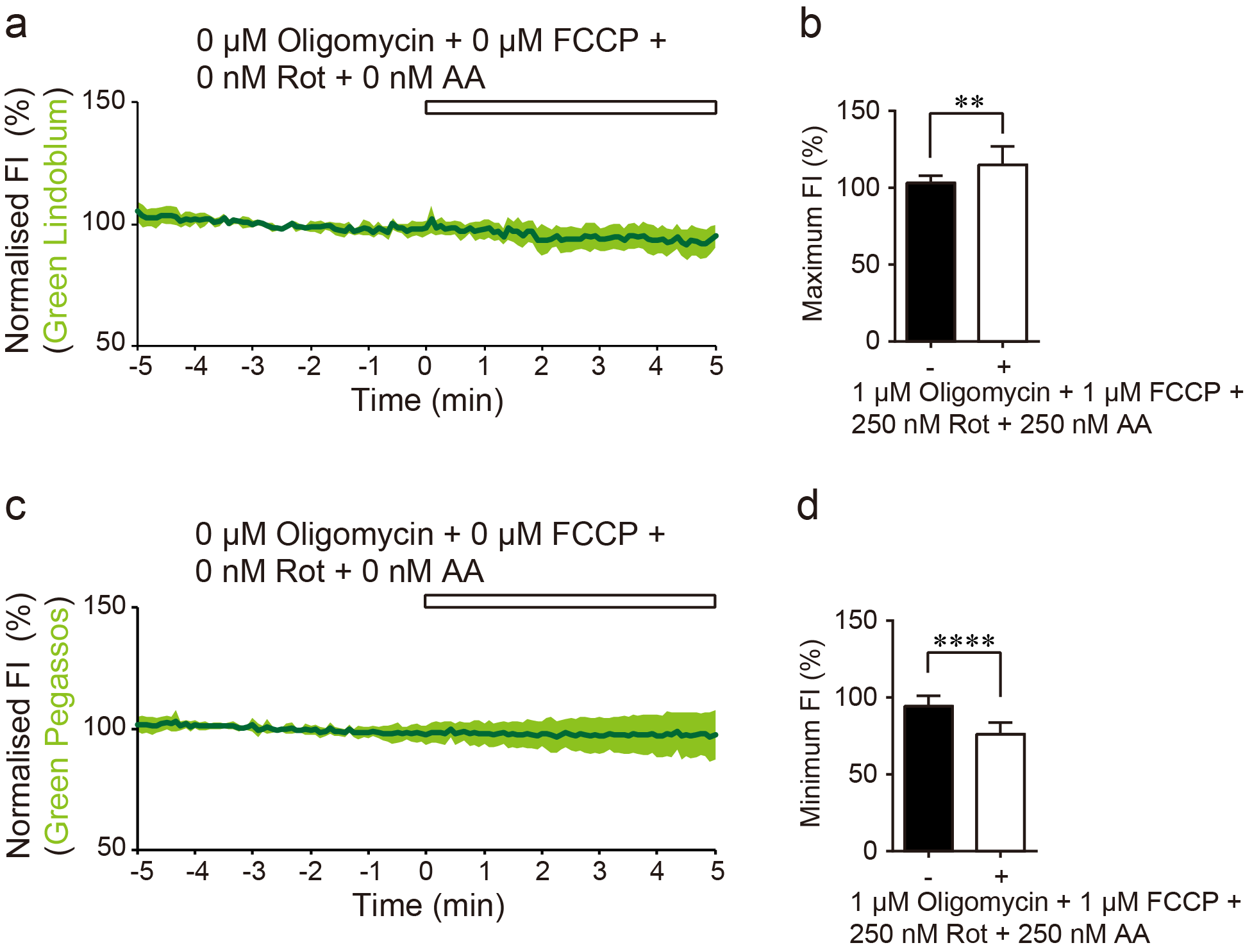


**Supplementary Figure S11. Imaging with human iPS cell-derived cardiomyocytes expressing Green Lindoblum and Green Pegassos without stimulation.** (**a**) Time course of the fluorescence intensity (FI) of Green Lindoblum in cardiomyocytes without inhibitors. (**b**) Comparison of maximum FI in Green Lindoblum in the presence (Fig. 4b) and the absence (Fig. S11a) of 1 μM oligomycin, 1 μM FCCP, 250 nM Rot, and 250 nM AA. (**c**) Time course of the fluorescence intensity (FI) of Green Pegassos in cardiomyocytes without inhibitors. (**d**) Comparison of maximum FI in Green Pegassos in the presence (Fig. 4c) and the absence (Fig. S11c) of 1 μM FCCP, 250 nM Rot, and 250 nM AA. The data are shown as means ± standard deviation (n = 7 (**a**) and 12(**c**), cells from three independent experiments). Welch’s *t* test. **, *P* < 0.01; ****, *P* < 0.0001.


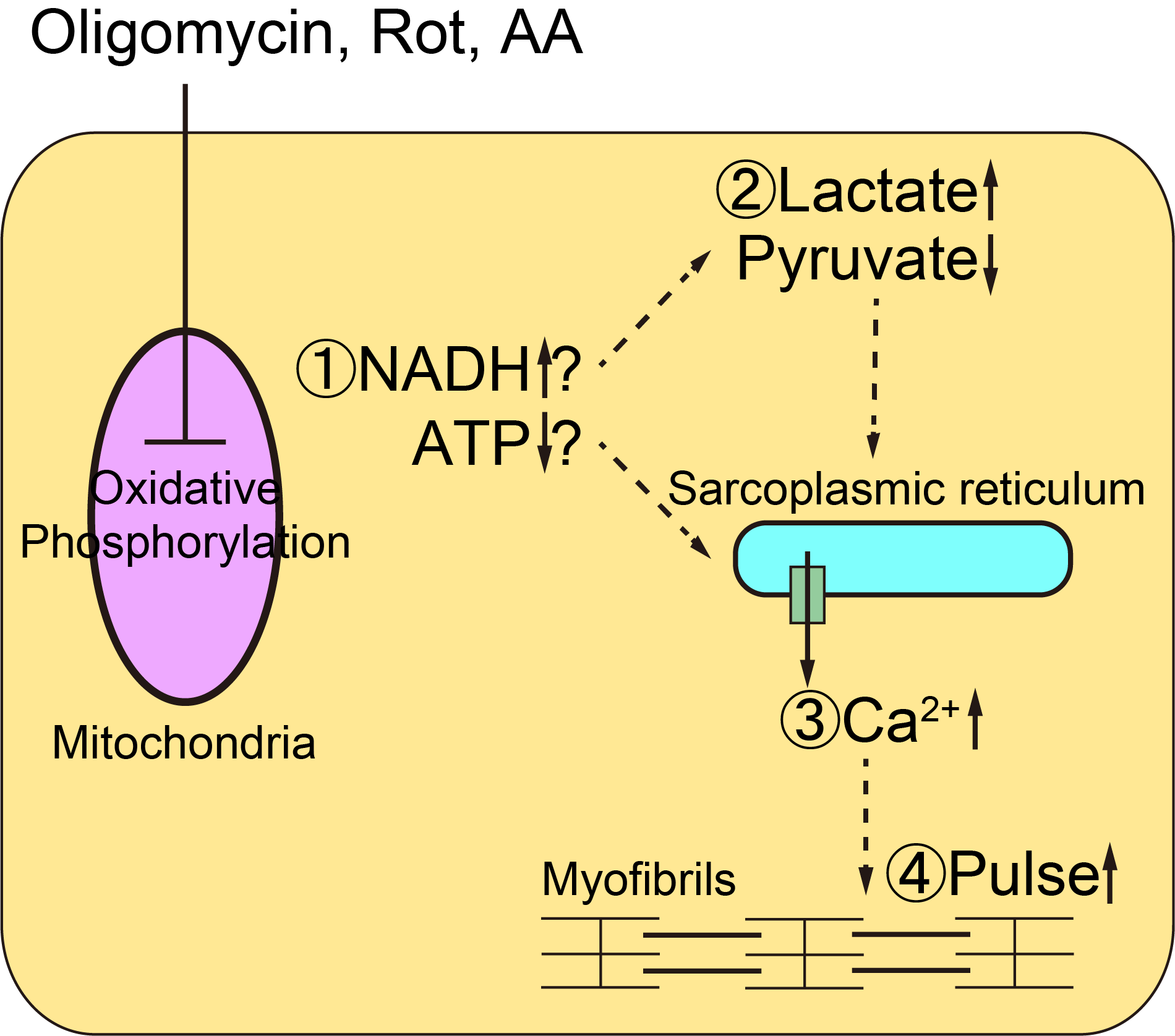


**Supplementary Figure S12. Schematic hypothesis based on the experiments with iPS cell-derived cardiomyocytes.** Inhibition of oxidative phosphorylation by oligomycin, Rot, and AA reduces ATP production in mitochondria and presumably causes NADH accumulation, although we could not observe directly in the present study (①). Perturbation of concentration balance between lactate and pyruvate (②), and malfunction of Ca^2+^-ATPase in the sarcoplasmic reticulum results in Ca^2+^ overload in the cytosol (③). Cytosolic Ca^2+^ elevation induces contraction of myofibrils and the increase of pulse frequency (④). However, in the long term cells exhaust ATP and pulse frequency declines.
